# BILBO: a self-hosted platform for large-scale RNA-Seq analysis with a functional annotation clustering framework

**DOI:** 10.1007/s10142-026-02091-9

**Published:** 2026-10-29

**Authors:** Vitor Luciano Costa da Silva, João Vitor Reis Alvarenga, Manoel Viana Linhares-Neto, Muhammad Noman, Antonio Chalfun-Junior

**Affiliations:** Institute of Natural Sciences, Department of Biology, Sector of Plant Physiology, Laboratory of Plant Molecular Physiology, Federal University of Lavras, Lavras, Minas Gerais Brazil

**Keywords:** Transcriptomics, DEG, Bioinformatics, Large language models

## Abstract

RNA sequencing (RNA-Seq) has become a central approach for transcriptomic studies, but its practical application still requires the coordination of multiple computational steps and often produces large gene expression outputs whose integrative biological interpretation remains challenging. Here, we present BILBO (Bioinformatics Integration for Large-scale Biological Operations), a self-hosted and fully containerized platform designed to support reproducible, end-to-end RNA-Seq analyses, from data acquisition to integrative functional interpretation. BILBO consolidates widely adopted open-source tools into standardized workflows, enabling automated retrieval of public or private RNA-Seq data, quality control, alignment, quantification, normalization, differential expression analysis, and generation of publication-ready outputs within a unified environment. Beyond workflow integration, BILBO introduces a novel functional annotation clustering framework that organizes genes using Gene Ontology (GO)-based semantic similarity. The approach separates genes by regulatory direction and ontology, computes Wang semantic similarity among GO annotations, applies complete-linkage hierarchical clustering, refines assignments through silhouette-based pruning, and selects representative genes as semantic medoids. To further enhance biological interpretability, BILBO integrates a Retrieval-Augmented Generation strategy that links representative genes and functional clusters to curated scientific literature through hybrid retrieval and DOI-linked evidence chunks. In a controlled SEQC/MAQC-III benchmark against nf-core/rnaseq, BILBO preserved differential-expression effect sizes (cross-workflow Spearman ≥ 0.994) and the expected behavior of the reference mixtures, whereas BILBO required 68% less peak temporary disk space but 25% more wall time. In the SEQC dataset, the resulting clusters showed strong coherence with independent MSigDB pathways, comparable to a GOSemSim-based reference workflow, and remained stable under moderate parameter changes. Together, these features position BILBO as an integrative and scalable solution that bridges the gap between large-scale RNA-Seq data processing and higher-level functional insight, promoting reproducibility, interpretability, and accessibility in transcriptomic research.

**Supplementary Information:**

The online version contains supplementary material available at https://doi.org/10.1007/s10142-026-02091-9.

## Introduction

Ribonucleic acid sequencing (RNA-Seq) has become a cornerstone of modern transcriptomics, enabling comprehensive and quantitative profiling of gene expression across a wide range of biological systems and experimental conditions (Million and Feyissa 2022). Advances in high-throughput sequencing technologies have dramatically increased data availability and reduced sequencing costs, leading to an unprecedented expansion of RNA-Seq applications in fields such as functional genomics, molecular medicine, plant biotechnology, and evolutionary biology (Todd et al. 2016; Lightbody et al. 2019; Nirmal et al. 2024). As a result, public repositories now host vast amounts of transcriptomic data, offering valuable opportunities for data reuse, comparative analyses, and meta-studies (Zoabi and Shomron 2021).

While RNA-Seq technologies have matured and become widely accessible, the practical execution of transcriptomic analyses still relies on the coordinated use of multiple software tools, reference datasets, and parameterized workflows (Lee et al. 2024). In practice, this fragmentation increases operational complexity and introduces variability across studies, particularly when analytical steps are executed in heterogeneous computational environments (Simoneau et al. 2021). As a consequence, researchers often face difficulties in maintaining consistent workflows and in efficiently transitioning from raw data processing to biological interpretation, especially in large-scale or comparative analyses.

In parallel with these technical challenges, the biological interpretation of RNA-Seq results remains a major bottleneck. While differential expression analyses routinely generate extensive gene lists, the downstream functional annotation and synthesis of biological meaning often rely on fragmented enrichment outputs, manual curation, or rigid ontology-based categorizations (Harbig et al. 2023; Oliveira et al. 2026). Conventional functional enrichment approaches tend to emphasize over-representation statistics but frequently fail to capture latent semantic relationships between gene functions, limiting their ability to reveal higher-order biological structure or emergent functional modules. Recent strategies, including similarity-based analyses and machine learning approaches leveraging embedding representations, have been proposed to uncover latent associations between gene functions and improve biological interpretation (Huang et al. 2025). In addition, emerging methods based on large language models have demonstrated the ability to infer biological patterns not detected by conventional enrichment approaches (Hu et al. 2025). However, these approaches remain insufficiently standardized and often lack interpretability or seamless integration with established annotation frameworks. This gap highlights the need for integrative strategies that move beyond gene-centric annotation toward data-driven functional organization.

To address these challenges, we present BILBO (Bioinformatics Integration for Large-scale Biological Operations), a self-hosted and fully containerized platform designed to support reproducible, large-scale RNA-Seq analyses from raw data acquisition to integrative functional interpretation. BILBO consolidates widely adopted open-source tools into standardized and automated workflows, enabling users to retrieve public sequencing data, perform quality control, alignment, quantification, normalization, and generate publication-ready outputs within a single, cohesive environment. By emphasizing accessibility, transparency, and portability, BILBO lowers technical barriers while promoting best practices in reproducible transcriptomics.

Beyond workflow integration, BILBO introduces a novel functional annotation clustering framework aimed at improving the interpretability of transcriptomic results. Rather than treating functional annotations as discrete or purely hierarchical labels, this approach organizes differentially expressed genes according to semantic relatedness among their Gene Ontology annotations. Crucially, this functional structuring is coupled with an automated biological interpretation layer based on Retrieval-Augmented Generation (RAG), which contextualizes clustered gene sets within the plant RNA-Seq literature. By embedding this strategy directly into the RNA-Seq analysis ecosystem, BILBO bridges the gap between large-scale expression profiling and higher-level functional insight, facilitating both exploratory and hypothesis-driven analyses. Moreover, its modular and literature-driven design enables adaptation to other kingdoms, supporting the interpretation of transcriptomic datasets across diverse biological systems, including animals, fungi, protists and prokaryotes.

Together, these features position BILBO as more than a pipeline manager: it is an integrative platform that combines standardized RNA-Seq processing with an innovative functional organization. By unifying computational rigor, reproducibility, and advanced functional interpretation within a self-hosted framework, BILBO contributes as a scalable solution for transcriptomic research and expands access to integrative genomics for researchers with diverse computational backgrounds.

## Materials and methods

### Data sources and acquisition

BILBO was designed to support automated and reproducible acquisition of transcriptomic data from public repositories. RNA-Seq datasets are retrieved directly from the National Center for Biotechnology Information (NCBI) Sequence Read Archive (SRA) using user-provided accession identifiers (Katz et al. 2022). Upon submission of valid SRA accessions, raw sequencing reads are automatically downloaded and converted into FASTQ format, making them immediately available for downstream analyses within the platform. This approach eliminates the need for manual data handling or external preprocessing steps, reducing potential sources of error and variability while streamlining data intake. Furthermore, BILBO also allows users to upload private FASTQ files directly to the platform, enabling the analysis of proprietary or unpublished RNA-Seq data without the requirement of public data deposition.

In addition to sequencing reads, BILBO enables the retrieval of reference genomes and their associated annotation files hosted by NCBI (O’Leary et al. 2024). Users may select any available reference genome relevant to their study, along with the corresponding gene annotation files (e.g., GFF format), which are subsequently incorporated into the analytical workflow. Reference sequences and annotations are stored locally and prepared for alignment and feature-level analyses in later stages of the pipeline, ensuring consistency between data acquisition and downstream processing.

### Pipeline components and third-party tools

The analytical backbone of BILBO is composed of widely adopted open-source bioinformatics tools that collectively support all major stages of RNA-Seq data processing, from raw read quality assessment to statistical analysis of gene expression. For initial quality control, raw sequencing reads are evaluated using FastQC, which provides detailed diagnostics on base quality scores, sequence composition, and potential technical artifacts prior to downstream processing (Simon Andrews et al. 2010). Adapter removal and quality trimming are performed with Trimmomatic, a flexible and multithreaded read-processing tool designed to improve read quality and alignment accuracy (Bolger et al. 2014). Read alignment to the reference genome is conducted using STAR (Spliced Transcripts Alignment to a Reference), a high-performance RNA-Seq aligner optimized for large datasets and accurate detection of splice junctions (Dobin et al. 2013). Following alignment, duplicate reads are identified and removed using Picard tools to minimize biases introduced by PCR amplification (Ebbert et al. 2016). Gene-level quantification is then performed with HTSeq, which counts reads overlapping annotated genomic features and produces raw expression count matrices suitable for statistical modeling (Anders et al. 2015).

Downstream normalization and differential gene expression analyses are carried out within the R statistical computing environment, ensuring a robust and widely accepted framework for transcriptomic data analysis. Specifically, BILBO integrates the edgeR package for normalization and inference, applying established methods based on negative binomial models to account for biological variability and differences in library size (Robinson et al. 2010). This integration allows statistically rigorous identification of differentially expressed genes while maintaining compatibility with community-standard RNA-Seq practices for reference genome-based analyses (Costa-Silva et al. 2023). All tools are orchestrated within a unified and automated pipeline (Supplementary File S1), with parameter configuration exposed through the BILBO interface, allowing users to define custom parameters while also providing predefined default settings as recommended starting points.

### Implementation details

BILBO was implemented as a modular system composed of backend services, a graphical user interface, and a persistent data layer. The application logic is primarily written in Python, with auxiliary scripts implemented in Shell and R for specific analytical tasks. The backend was developed using the FastAPI framework and exposes RESTful endpoints for all stages of the RNA-Seq workflow. Backend services handle request routing, parameter validation, execution control, and state tracking of analytical steps. Computational tasks are executed via subprocess calls to Shell, Python, or R scripts, depending on the requirements of each processing stage. Asynchronous execution is implemented using the async/await paradigm to allow concurrent task handling.

Persistent data storage is managed through a PostgreSQL relational database accessed via SQLAlchemy as an object–relational mapping (ORM) layer. The database schema stores user accounts, sample metadata, analysis stages, and execution status information. Database sessions are handled transactionally to maintain consistency across pipeline executions.

The user interface was developed in Python using the Flet framework and is accessible through a web browser. The interface is structured into functional modules corresponding to individual stages of the RNA-Seq pipeline, including data registration, quality control, alignment, quantification, differential expression analysis, and visualization. Communication between the frontend and backend is performed using asynchronous HTTP requests and WebSockets.

The system is deployed using Docker, with services encapsulated in isolated containers (Acharya and Suthar 2022). Container orchestration is managed via docker-compose, which defines the execution environment for the backend and database services, as well as the required software dependencies. Persistent volumes are used to store user data and analysis outputs. Remote access to the platform is provided through SSH local port forwarding, where the server-side application port is exposed to the client machine using secure tunneling (e.g., ssh -L). User authentication is implemented using OAuth2 with JSON Web Tokens (JWT), and access to protected endpoints requires token validation.

### Comparative benchmarking against nf-core/rnaseq

The reference-based RNA-seq core of BILBO was benchmarked against nf-core/rnaseq v3.26.0, selected as a mature, community-curated, containerized workflow with directly comparable input and output scope (Ewels et al. 2020). The benchmark used paired-end SEQC/MAQC-III libraries from GEO series GSE47774 (SEQC/MAQC-III Consortium 2014). The design comprised four replicates each of reference samples A (Universal Human Reference RNA) and B (Human Brain Reference RNA), together with the known mixtures C (75% A + 25% B) and D (25% A + 75% B), for a total of 16 samples. Subsets of 1 and 5 million read pairs per sample were generated from read-pair identifiers. Data acquisition and subsampling were common to both workflows and were excluded from the timed regions. Both workflows used the same *Homo sapiens* GRCh38 primary assembly and Ensembl release 112 annotation and were configured with ceilings of 128 logical CPUs and 128 GB RAM on the same server.

The comparison was designed as an end-to-end workflow benchmark rather than a test of identical component algorithms. The evaluated BILBO build used FastQC, Trimmomatic, STAR, Picard, HTSeq-count and edgeR, whereas nf-core/rnaseq was executed with the “star_salmon” alignment/quantification route, Trim Galore and its Docker profile. nf-core modules without a functional counterpart in BILBO were disabled. For both workflows, the timed region included reference-index generation, quality control, trimming, alignment, post-alignment processing, gene-level quantification and report generation. One technical run per workflow was performed at 1 million read pairs per sample, and two technical runs per workflow were performed at 5 million read pairs per sample, with workflow order reversed in the second 5-million-pair run to reduce order-related bias. The first nf-core/rnaseq 1-million-pair run recorded initial retrieval of the workflow source and the nf-schema plugin and was consequently treated as a transparent cold-start observation rather than the primary runtime comparison. Comparative performance interpretation was based on the two warm-cache 5-million-pair runs.

Biological validity was evaluated independently of either workflow. RNA-seq expression was compared with TaqMan RT-PCR measurements from the Bioconductor “seqc” experimental data package, and the observed expression of mixtures C and D was compared with the expected 75:25 and 25:75 combinations of A and B. Duplicate TaqMan assay rows sharing a gene symbol were collapsed before comparison by taking the median quantitative value and the majority detection call for each replicate. Differential expression between A and B was evaluated in two ways: using the statistical procedure implemented in BILBO and using a common robust edgeR quasi-likelihood analysis applied independently to both standardized count matrices. Cross-workflow agreement was quantified using gene-level log2-counts-per-million correlations, Spearman correlation of log2 fold changes, concordance of effect direction and the Jaccard index of genes meeting FDR < 0.05 and |log2 fold change| ≥ 1. Wall time, peak memory and peak disk-space increase were recorded from the complete containerized executions. The complete design, numerical results and limitations are provided in Supplementary File S2.

### Functional annotation clustering

Functional annotation clustering was implemented as a multi-stage computational procedure designed to group genes based on the semantic relatedness of their Gene Ontology annotations. Functional descriptors associated with each gene were collected from Gene Ontology records and UniProt annotation sources generated during the annotation stage of the pipeline (The Gene Ontology Consortium 2026; The UniProt Consortium 2025).

To strengthen the biological basis of the clustering procedure, BILBO implements an ontology-grounded semantic similarity strategy based on Gene Ontology (GO) annotations. Rather than relying exclusively on lexical proximity among textual annotations, this workflow evaluates functional relatedness directly from the GO directed acyclic graph using the Wang semantic similarity method, which quantifies the semantic contribution of GO terms and their ancestor terms within the ontology structure (Wang et al. 2007). Semantic similarity calculations were performed using the GOSemSim framework, which supports similarity estimation among GO terms, gene products, and gene clusters (Yu et al. 2010). The procedure is applied independently to up- and down-regulated genes, so that genes with opposite regulatory behavior are not grouped solely because they share broad functional annotations. In addition, clustering is performed separately for the three GO ontologies: biological process (BP), molecular function (MF), and cellular component (CC). This separation preserves the biological meaning of each ontology: BP clusters emphasize shared processes, MF clusters emphasize shared molecular activities, and CC clusters emphasize shared subcellular localization or compartment association.

For each ontology * o* in BP, MF or CC, the annotations associated with gene * i* are represented as a set of valid GO terms, denoted as $\:{G}_{i}^{\left(o\right)}$. Invalid GO identifiers are removed, whereas obsolete terms are mapped to their current valid counterparts using the Gene Ontology resource, ensuring that all pairwise comparisons are computed against a consistent ontology structure (The Gene Ontology Consortium 2026). Genes without sufficient valid terms in a given ontology are retained in the output but are not forced into a cluster for that ontology.

The functional similarity between two genes is calculated using the Wang semantic similarity over the GO directed acyclic graph. If $\:W\left(g,h\right)$ denotes the Wang similarity between GO terms * g* and * h*, the average best-match score from gene * i* to gene * j* is defined as:

$$\:A\left({G}_{i}^{\left(o\right)},{G}_{j}^{\left(o\right)}\right)=\frac{1}{\left|{G}_{i}^{\left(o\right)}\right|}{\sum_{g\in\:{G}_{i}^{\left(o\right)}}}\underset{{\:\:\:h}\in\:{G}_{j}^{\left(o\right)}}{\mathrm{max}}W\left(g,h\right)$$

Because the best-match relationship is directional, the final gene-level Wang similarity is obtained by averaging both directional scores:

$$\:{S}_{ij}^{\left(o\right)}=\frac{1}{2}\left[A\left({G}_{i}^{\left(o\right)},{G}_{j}^{\left(o\right)}\right)+A\left({G}_{j}^{\left(o\right)},{G}_{i}^{\left(o\right)}\right)\right]$$

This formulation assigns each gene pair a bounded semantic similarity score, where values closer to 1 indicate stronger functional relatedness within the selected ontology. The similarity matrix is then transformed into a distance matrix:

$$\:{d}_{ij}^{\left(o\right)}=1-{S}_{ij}^{\left(o\right)}$$

Genes are grouped using agglomerative hierarchical clustering with complete linkage, a conservative clustering strategy that favors compact groups and reduces the chaining effect commonly observed in hierarchical clustering (Murtagh and Contreras 2012). For two candidate clusters $\:{C}_{a}$ and $\:{C}_{b}$, their distance is defined as the largest pairwise distance between their members:

$$\:D\left({C}_{a},{C}_{b}\right)=\underset{i\in\:{C}_{a},j\in\:{C}_{b}}{\mathrm{max}}{d}_{ij}^{\left(o\right)}$$

Complete linkage was selected because it favors clusters in which all members maintain a minimum level of mutual similarity. This choice is particularly suitable for functional annotation clustering, since it reduces the probability that broad modules are formed through only one highly similar gene pair.

After initial clustering, small or poorly supported groups are treated conservatively. Clusters containing fewer than the minimum required number of genes are assigned as unclustered. The remaining assignments are evaluated using the silhouette coefficient, which provides an internal measure of how well each gene fits its assigned cluster relative to alternative clusters (Rousseeuw 1987):

$$\:s\left(i\right)=\frac{b\left(i\right)-a\left(i\right)}{\mathrm{max}\left(a\left(i\right),b\left(i\right)\right)}$$

where $\:a\left(i\right)$ is the mean distance between gene * i* and the other genes in its assigned cluster, and $\:b\left(i\right)$ is the smallest mean distance between gene * i* and genes in any other cluster. Genes with negative silhouette values are iteratively removed from their assigned clusters, because such values indicate that the gene is, on average, closer to another group than to the cluster in which it was initially placed. For each retained cluster, BILBO reports multiple quality metrics to support downstream inspection, including individual silhouette values, mean silhouette, minimum silhouette, cluster size, minimum pairwise similarity, and a categorical cluster quality label. Genes that do not pass the clustering or validation criteria remain in the output with cluster assignment (− 1), preserving transparency rather than forcing every gene into a functional module.

A representative gene is selected for each retained cluster to provide a compact biological entry point for downstream interpretation. Instead of relying on a purely geometric centroid, the representative gene is defined as the most central member of the cluster according to its mean Wang similarity to all other genes in the same module. For a cluster * C*, the semantic centrality of gene * i* is calculated as:

$$\:{c}_{i}=\frac{1}{\left|C\right|-1}{\sum_{j\in\:C,j\ne\:i}}{S}_{ij}$$

The gene with the highest $\:{c}_{i}$ value is selected as the semantic medoid of the cluster. Equal centrality scores are resolved using cluster silhouette, absolute log2 fold change and gene identifier ordering. Representative genes supported across multiple ontologies are prioritized for literature retrieval, because their centrality is reinforced by independent BP, MF, or CC evidence rather than by a single annotation view.

The clustering procedure was evaluated against a reference workflow implemented with GOSemSim v2.32.0 (Yu et al. 2010). Both workflows used the same genes, GO annotations, Wang best match average similarities, thresholds defined for each ontology, and a minimum group size of three genes. In the reference workflow, the similarity matrix was clustered using average linkage, and all genes in groups of the required size were retained. It did not apply the subsequent BILBO silhouette filter, which removes genes that fit another cluster better than the group to which they were initially assigned. Sensitivity was assessed by changing one component at a time, including the linkage method, GO similarity thresholds, and silhouette cutoff. Similarity to the cluster assignments produced with the settings used by BILBO was quantified with the adjusted Rand index (ARI), where one denotes identical assignments and values near zero indicate little similarity beyond chance.

Functional coherence was evaluated using the MSigDB C2:CP: KEGG_MEDICUS pathway collection (Subramanian et al. 2005; Liberzon et al. 2011). Because the clusters were derived from GO similarities, MSigDB provided a distinct biological reference for this evaluation. After both workflows had been applied, the direct comparison was restricted to the 2,262 genes that received a Biological Process cluster assignment from both methods. For each cluster, we calculated the proportion of gene pairs sharing at least one MSigDB pathway and then averaged these proportions across clusters, giving equal weight to each cluster so that larger groups did not dominate the result. As a complementary analysis, pathway over-representation was assessed for each cluster using a hypergeometric test, with Benjamini–Hochberg correction across the tested pathways (FDR < 0.05 were considered significant). As a negative control, genes were randomly reassigned to groups while preserving the number of genes per cluster, the direction of differential expression, and the density of GO annotations. This control estimated how much pathway sharing could arise from these structural characteristics alone rather than from the functional relationships captured by the clustering methods. To examine whether the results depended on extensively annotated genes, the analysis was repeated separately for genes annotated with up to 12 GO terms and for genes with more than 12 terms, corresponding to the median annotation count in the evaluated set. Uncertainty in the difference between methods was estimated from 5,000 bootstrap resamples of the clusters.

To bridge the gap between functional grouping and biological meaning, BILBO integrates the clustering output directly with a Retrieval-Augmented Generation (RAG) module. This integration allows representative genes and their functional cluster context to be interpreted against a curated scientific literature corpus, setting the stage for the interpretation phase described in the subsequent section.

### Automated biological interpretation via Retrieval-Augmented Generation (RAG)

To overcome the limitations of manual curation and the potential for factual hallucinations in conventional Large Language Models (LLMs), BILBO implements a Retrieval-Augmented Generation (RAG) architecture (Zhao et al. 2026). This module decouples the model’s reasoning capabilities from its internal knowledge base, dynamically connecting it to a curated corpus of scientific literature. This corpus was constructed from 1,612 studies indexed in PlantExp (Liu et al. 2023), a platform dedicated to the exploration of gene expression and alternative splicing based on publicly available plant RNA-seq datasets, ensuring that all retrieved documents are directly associated with transcriptomic experiments. The plant-specific corpus generated for this study is publicly available at Zenodo (https://zenodo.org/records/21855338), and the corresponding DOIs of all source articles are provided in Supplementary File S3.

The literature ingestion step processes scientific articles in Markdown format by section. Low-information sections, including references, funding statements, methods-only fragments, figure captions, tables, and table-like chunks, are excluded from the retrieval index when they are unlikely to support functional interpretation. The remaining text is divided into sentence-aware chunks of approximately 350 words, with an overlap of two complete sentences between adjacent chunks. This strategy preserves syntactic and biological context across boundaries while reducing the risk of mixing unrelated article sections within the same retrieval unit. Each chunk is stored with source metadata, including article file, article title, section title, section index, chunk index, word count, and a stable chunk identifier. During indexing, chunks are also annotated for biological signals, including gene-like mentions, species mentions, cellular components, simple chemicals, protein families, and GO terms. These metadata fields are later used as explicit retrieval and reranking signals, allowing the system to distinguish direct gene or alias evidence from broader family-level, ontology-level, or process-level context.

BILBO indexes each chunk in Qdrant using a hybrid representation. The dense component is produced with the BGE-M3 embedding model, which maps each chunk into a vector representation for semantic retrieval and was selected because of its multi-functionality and multi-granularity design for retrieval tasks (Chen et al. 2024). In parallel, a local BM25 model generates a sparse vector that preserves exact lexical evidence, such as gene aliases, protein family names, and ontology terms. This dual representation allows the system to recover both semantically related passages and passages containing exact terminology matches.

For a query * q* and document chunk * d*, the sparse retrieval component follows the BM25 scoring function (Robertson and Zaragoza 2009):

$$\:BM25\left(q,d\right)={\sum_{t\in\:q}}IDF\left(t\right)\cdot\:N\left(t,d\right)$$

$$\:N\left(t,d\right)=\frac{f\left(t,d\right)\left({k}_{1}+1\right)}{f\left(t,d\right)+{k}_{1}\left(1-b+b\frac{\left|d\right|}{avgdl}\right)}$$

where $\:f\left(t,d\right)$ is the frequency of term * t* in chunk * d*, $\:\left|d\right|$ is the chunk length, * avgdl* is the mean chunk length in the indexed corpus, and $\:{k}_{1}$ and * b* control term-frequency saturation and length normalization, respectively. The dense component uses cosine similarity between query and chunk embeddings:

$$\:sim\left(q,d\right)=\frac{e\left(q\right)\cdot\:e\left(d\right)}{\left|e\left(q\right)\right|\left|e\left(d\right)\right|}$$

The retrieval query is deliberately separated into complementary components. The sparse BM25 query is built from gene metadata, including the primary gene name, GFF-derived name, UniProt gene names, parenthetical aliases, and compact symbol-like aliases. In contrast, the dense embedding query is built from the functional context associated with the representative gene, especially ontology-derived terms linked to its cluster. This separation prevents semantic retrieval from being dominated by gene symbols alone while preserving exact alias matching through BM25.

Dense and sparse retrieval results are fused in Qdrant using reciprocal rank fusion (RRF), a rank aggregation method designed to combine outputs from multiple retrieval systems (Cormack et al. 2009). If $\:{r}_{m}\left(d\right)$ is the rank of document * d* under retrieval method * m*, the fused score is defined as:

$$\:RRF\left(d\right)={\sum_{m}}\frac{1}{k+{r}_{m}\left(d\right)}$$

The resulting candidate chunks are then passed through a local reranking step that favors evidence containing gene or alias matches, protein-family matches, GO-term matches, and contextual biological terms either in the chunk text or in the annotated metadata payload. To reduce redundancy, the final selection limits the number of chunks that can be retrieved from the same article source before diversifying across sources. Low-value chunks are filtered again at retrieval time, and each selected chunk receives a citation identifier, such as C1, C2, or C3. For each retrieval event, the system records observational audit signals, including the number of returned chunks, number of independent sources and sections, chunks containing biological payload signals, observed gene or alias matches, protein-family matches, GO-term matches, and contextual terms. These fields are not treated as categorical evidence levels, but as descriptive indicators that allow users to inspect the strength and nature of the retrieved support.

The final synthesis is executed locally using the Gemma 4 E4B model, orchestrated via the Ollama framework. The model receives only the gene metadata, cluster-level functional context, and retrieved chunks. A closed-knowledge constraint is imposed through the system prompt, requiring that biological claims be derived exclusively from the provided chunks or explicitly identified as unsupported when the retrieved literature is insufficient. This design reduces the risk of hallucination by transforming the model from an open-ended generator into a constrained evidence synthesizer. The model is required to return a structured JSON with three main fields, that is subsequently organized into HTML reports. The chunk_interpretations field contains one item per retrieved chunk, summarizing the supported observation, the relevance of that chunk to the gene, the basis of the match, and the limitation of the evidence. The cross_chunk_synthesis field integrates only observations supported by chunk-level reading. The final_interpretation field provides a concise biological interpretation for the representative gene, with literature-derived claims cited using the chunk identifiers, for example [C1] or [C1, C2]. As a demonstrative case study within the present manuscript, representative genes selected from the GO-based functional clusters were submitted to the RAG interpretation module. The corresponding tabular summaries, including representative genes selected for RAG analysis and expert review notes, are provided in Supplementary File S4.

Retrieval performance was evaluated using a frozen set of 13 gene queries and the complete corpus of 53,037 literature chunks. BM25, BGE-M3, hybrid BM25 + BGE-M3 retrieval, BioBERT (Lee et al. 2020), and PubMedBERT (Gu et al. 2021) were compared using the same queries, corpus, retrieval depth, and post-processing procedure. For each query, the ten highest-ranked passages from each method were pooled and deduplicated before expert assessment, with method identity and rank concealed during annotation. Relevance was classified as direct, indirect or contextual, or irrelevant. Performance was summarized using Precision@5, graded nDCG@10, reciprocal rank of the first directly relevant passage, and pooled Recall@10. The latter was calculated within the judged pool because exhaustive relevance assessment of all corpus passages was not feasible. BioBERT and PubMedBERT were evaluated using attention-mask mean pooling, L2 normalization, 512-token truncation, and cosine similarity.

The interpretations generated in the final evaluation run were subsequently examined against their cited passages by a domain expert. Each atomic claim was classified as supported, partially supported, unsupported, or contradicted, and citation correctness was assessed separately. Claim support was reported together with answer coverage and abstention, thereby accounting for genes for which the workflow withheld an interpretation because the available evidence was insufficient. Detailed methods and results are provided in Supplementary File S5. Runtime benchmarks for the GO-based functional clustering and RAG-assisted interpretation modules across DEG datasets of different sizes are provided in Supplementary File S6. The full experimental design, numerical results, and limitations of the comparative validation of functional clustering are provided in Supplementary File S2.

This structure makes the RAG output directly auditable. Users can inspect whether a conclusion is based on direct gene or alias evidence, protein-family evidence, GO or process context, or only weak background information. When the retrieved chunks do not support a gene-specific conclusion, the system is instructed to state this limitation rather than infer beyond the available evidence.

## Results and discussion

### System architecture and workflow overview

**Fig. 1:**
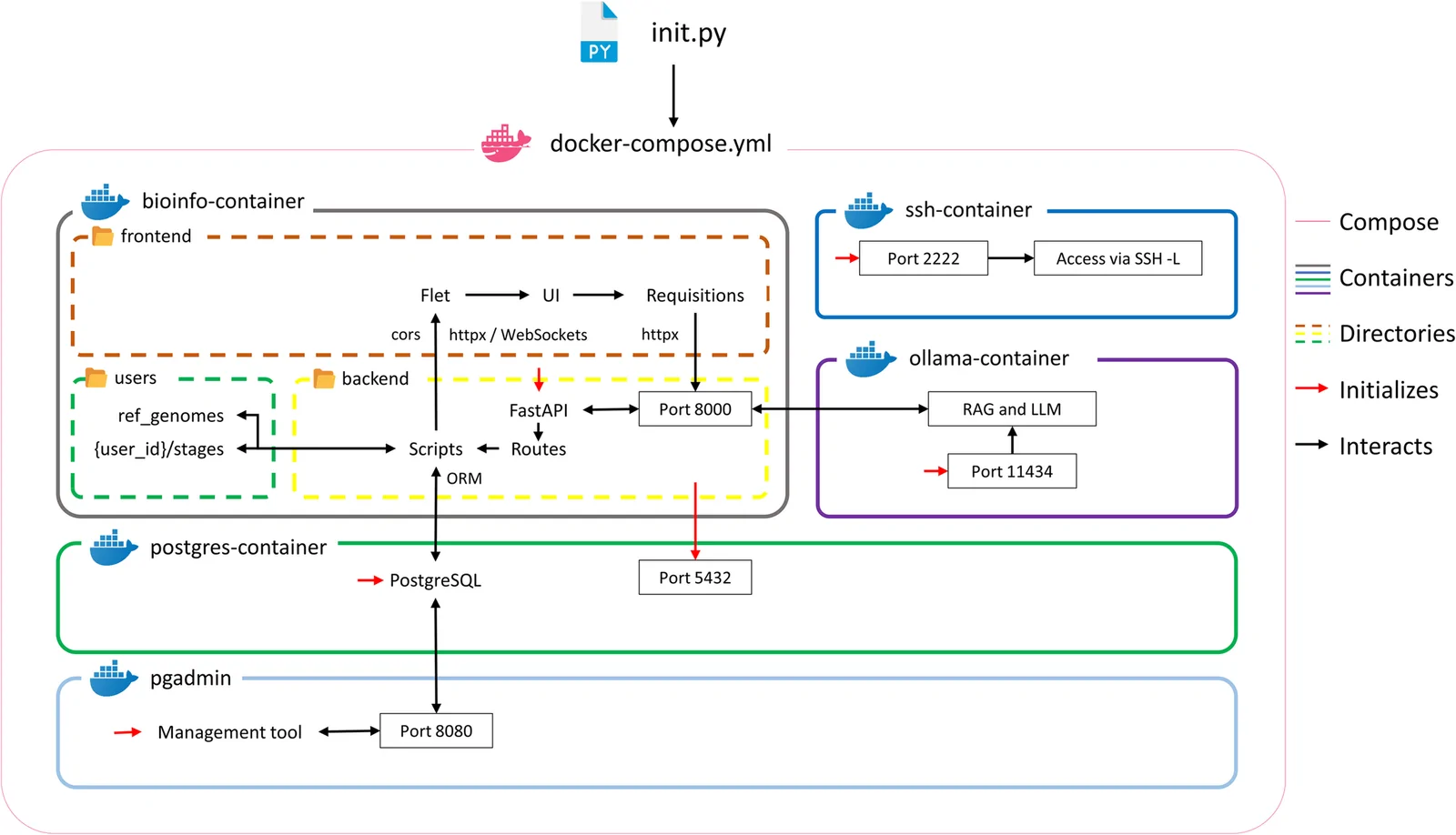
General architecture of the system and inter-container workflow. Arrows indicate interactions (black) and initialization commands (red). Directory boundaries are marked with dashed lines, while container boundaries are shown with solid colored lines

The system combines backend services, a web-based user interface, auxiliary processing scripts, and a persistent data layer, enabling coordinated execution of complex analytical workflows while maintaining clear separation of responsibilities between components (Fig. 1).

Backend services orchestrate the RNA-Seq pipeline by managing user authentication, request validation, execution control, and tracking of analysis states. Each analytical stage, from raw data retrieval to differential expression analysis, is executed as an independent module with explicit status management, allowing monitoring of pipeline progression. Computational tasks are delegated to external scripts written in Shell, Python, and R, which are executed asynchronously and in isolation, enabling parallel processing and fault containment without interrupting the overall workflow.

**Fig. 2:**
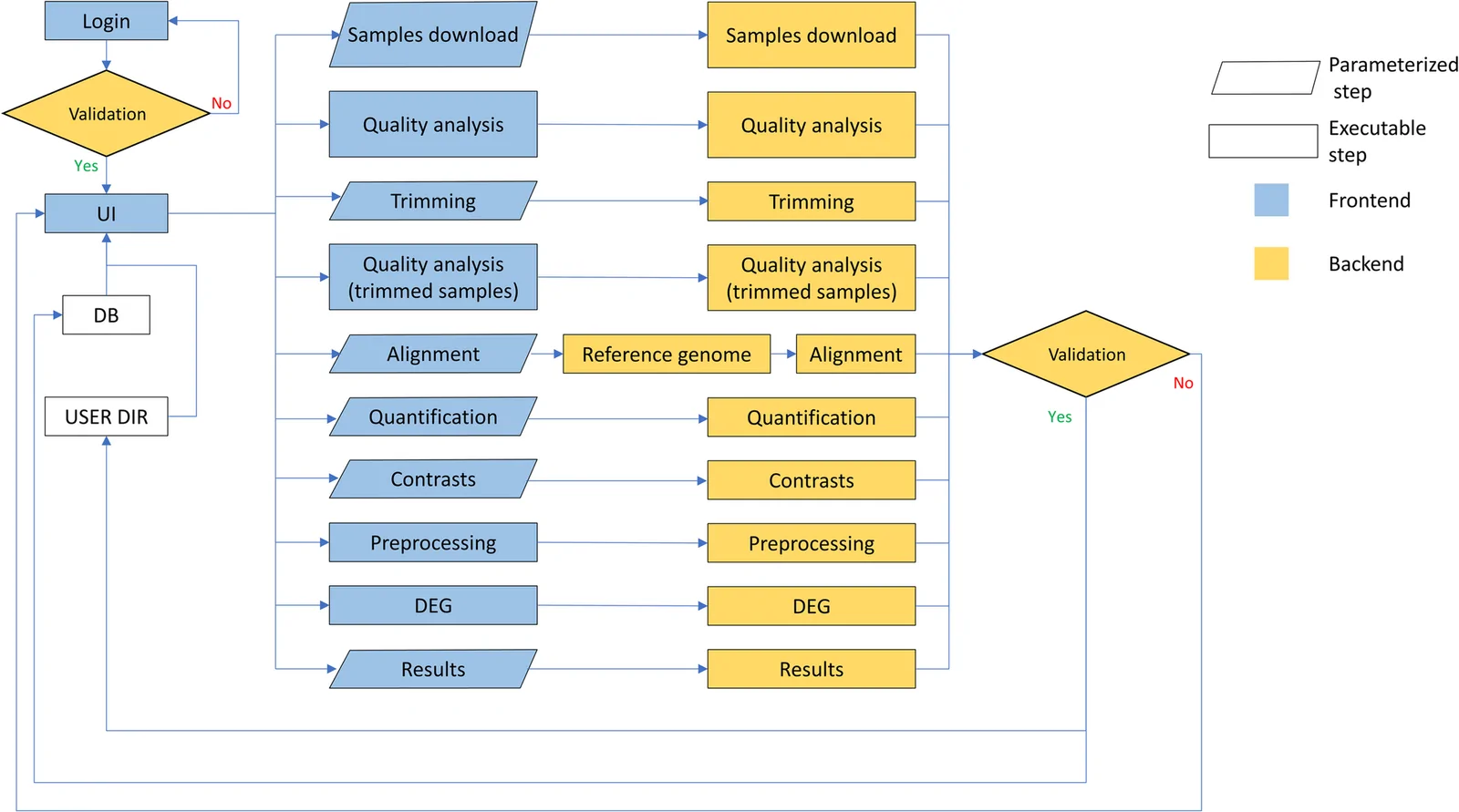
Overview of the RNA-Seq analysis workflow and interaction between system components. Blue elements represent frontend components, where the user initiates each step and interacts with the system via graphical elements. Yellow elements correspond to backend processes, where computational tasks are validated and executed. White boxes represent external resources, such as the database (DB) and user-specific directories (USER DIR), used for storing metadata and results

User interaction with the system occurs through a browser-accessible graphical interface that exposes all pipeline stages as discrete, sequential steps. Through this interface, users can register samples, configure analysis parameters, initiate processing tasks, and inspect intermediate and final results without requiring command-line interaction. The workflow design balances sequential execution with flexibility, allowing individual stages to be re-executed or adjusted as needed. The RNA-Seq workflow is organized as a sequence of frontend–backend interaction cycles, in which each analytical stage is triggered by user actions, validated by backend services, and executed through dedicated processing modules (Fig. 2). If validation fails at any stage, execution is halted and the system redirects the user to the interface with feedback, allowing parameter correction without advancing the workflow.

The adoption of a centralized data layer allowed analysis states and intermediate results to remain accessible across execution steps, enabling users to revisit, reconfigure, or extend analyses without repeating the entire workflow. Combined with container-based execution, this design supported stable operation across different computational environments and facilitated systematic execution of RNA-Seq analyses. In practice, these architectural decisions contributed to a streamlined analytical process in which pipeline stages could be monitored and managed coherently, reinforcing BILBO’s suitability for end-to-end transcriptomic data analysis.

### End-to-end RNA-Seq analysis using BILBO

BILBO executes the RNA-Seq analysis pipeline as a sequence of backend-triggered computational stages, each launched via Python’s subprocess module using either subprocess.run or subprocess.Popen. This execution model enables asynchronous processing, real-time log generation, and continuous status updates stored in the PostgreSQL database, allowing users to monitor pipeline progression during execution.

Sample acquisition is performed using the SRA Toolkit through the fasterq-dump command, wrapped in the download_script.sh Bash script. Each download task is executed within an independent tmux session, which allows persistent execution and parallel downloads across samples. Sample-specific log files are generated during this step, providing detailed traceability of download progress and facilitating error diagnosis when required. Reference genome retrieval is handled separately using the NCBI Datasets CLI, invoked via download_genome.sh, which automatically organizes downloaded files by retaining the reference FASTA sequence and renaming the main annotation file to genomic.gff for downstream compatibility.

Read preprocessing is performed using Trimmomatic through the trimmagem.sh script. During execution, the script automatically detects whether the input data correspond to single-end or paired-end sequencing libraries and adjusts trimming parameters accordingly. Phred quality score encoding is inferred automatically, and custom adapter files can be incorporated when provided by the user. Following trimming, post-processing quality control is carried out using FastQC via quality_analysis_post_trim.sh, ensuring that trimmed reads meet quality requirements prior to alignment.

Read alignment is conducted using STAR, executed through the alignment.sh script. This step prepares temporary working directories, applies user-defined alignment parameters such as thread count and indexing options, and generates BAM alignment files. Post-alignment processing is performed using Picard tools to sort alignments, assign read groups, and remove duplicate reads. As in the download stage, alignment tasks are executed within dedicated tmux sessions, enabling isolated execution and detailed monitoring of long-running processes.

Gene-level quantification is performed using HTSeq-count through the quantification.sh script. This stage validates the presence and integrity of the required BAM and GFF files, applies the appropriate feature type and gene identifier parameters, and generates tab-delimited count files for each sample. These count matrices constitute the primary input for downstream statistical analyses.

Preprocessing and differential gene expression (DEG) analyses are carried out in the R environment using the preprocess.R and DEG.R scripts, respectively. The preprocessing step employs the edgeR package to perform TMM normalization, low-count filtering, and exploratory data analysis. DEG analysis applies negative binomial modeling, dispersion estimation, generalized linear model fitting, and statistical testing to identify differentially expressed genes, with results exported as Excel spreadsheets for downstream interpretation.

Throughout pipeline execution, all input and output files are organized within structured, stage-specific directories assigned per user. Each analytical step includes parameter validation, directory checks, and logging procedures to ensure consistency across executions. Upon completion of each stage, outputs are automatically registered and made available for visualization within the frontend, allowing users to inspect intermediate and final results while preserving traceability across the entire RNA-Seq workflow.

### Output visualization and interpretation tools

BILBO provides a set of integrated visualization and exploration tools designed to support direct interpretation of RNA-Seq results within the platform. For each stage of the analytical workflow, dynamically generated graphical and tabular outputs are produced using custom Python and R scripts, allowing users to inspect data quality, explore expression patterns, and interpret differential expression results without exporting intermediate files or relying on external software.

During quality control, FastQC is applied to both raw and trimmed sequencing reads, and the resulting HTML reports are automatically parsed and embedded into the frontend. This integration allows interactive inspection of standard quality metrics, including per-base sequence quality, GC content, and duplication levels, directly within the user interface. Access to both pre- and post-trimming reports enables users to evaluate the effects of adapter removal and quality filtering and to identify residual technical artifacts before alignment.

For normalization and exploratory data analysis, visualization modules are generated from edgeR outputs produced during preprocessing. These include library size barplots, multidimensional scaling (MDS) plots, sample clustering visualizations, and count density distributions. The interface allows users to switch interactively between these plots through selection controls, facilitating exploratory assessment of sample relationships, detection of outliers, and evaluation of technical variability across libraries.

Differential expression analysis results are accompanied by standard and widely adopted visual summaries. Automatically generated barplots report the number of up- and down-regulated genes between experimental conditions, while MA plots and volcano plots provide complementary views of expression magnitude and statistical significance. These visualizations enable rapid identification of strongly regulated genes and global expression trends, supporting both exploratory and confirmatory analyses.

In addition to graphical summaries, BILBO integrates tabular exploration of results with functional annotation metadata. Differential expression tables include gene identifiers, log fold changes, expression values, and statistical measures, and support interactive filtering, sorting, and keyword-based searches. Gene identifiers are linked to annotation information derived from GFF files and UniProt records, enabling retrieval of gene descriptions, ontology terms, and functional summaries (Supplementary File S7). The RNA-seq libraries used in this analysis were originally generated by de Oliveira et al. (2020) and are available in the SRA under BioProject PRJNA609253. This integration allows users to move seamlessly from quantitative expression results to biological interpretation within a single environment. All graphical outputs are automatically saved in high-resolution formats suitable for publication and can be retrieved for downstream use.

### Computational performance relative to nf-core/rnaseq

Both workflows completed the controlled SEQC/MAQC-III benchmark and produced structurally valid matrices containing the same 63,140 gene identifiers across all 16 samples. BILBO produced lower total gene counts than nf-core/rnaseq, with median BILBO-to-nf-core ratios of 0.784 at 1 million and 0.751 at 5 million read pairs per sample. This difference was expected because the workflows use fundamentally different quantification strategies: BILBO assigns genome-aligned reads directly to genes using HTSeq, whereas nf-core/rnaseq estimates transcript abundance with Salmon and subsequently aggregates transcript-level estimates to genes using tximport. Previous benchmarking has shown that alignment-based gene-counting methods such as HTSeq may assign fewer reads and underestimate expression relative to Salmon (Zheng et al. 2019). After library-size normalization, the median per-sample Pearson correlation of log_2_(CPM + 1) values was 0.921 at 1 million and 0.923 at 5 million read pairs. Importantly, the inferred biological effects were highly concordant. Across the common robust edgeR quasi-likelihood and BILBO integrated statistical analyses, cross-workflow Spearman correlations of A-versus-B log_2_ fold changes ranged from 0.9944 to 0.9961, while the Jaccard index of differentially expressed gene sets ranged from 0.8223 to 0.8580.

External validity was assessed using two complementary SEQC reference measurements. At 5 million read pairs per sample, correlations between RNA-seq expression and TaqMan measurements across samples A–D ranged from 0.752 to 0.836 for BILBO and from 0.802 to 0.871 for nf-core/rnaseq. Agreement of the A-versus-B log_2_ fold change with TaqMan was nearly identical (Spearman 0.963 for BILBO and 0.967 for nf-core/rnaseq; median absolute error 0.394 and 0.388, respectively). Agreement with the expected expression profiles of the SEQC mixtures was also high. For mixtures C and D, respectively, the Spearman correlations were 0.9951 and 0.9959 for BILBO and 0.9947 and 0.9954 for nf-core/rnaseq. The corresponding log_2_-scale RMSE values were 0.1976 and 0.1817 for BILBO and 0.2093 and 0.1998 for nf-core/rnaseq. Across the TaqMan- and mixture-based evaluations, both workflows closely reproduced the independent SEQC reference patterns, with only small numerical differences among the observed metrics.

Computational performance was assessed primarily in the replicated warm-cache condition at 5 million read pairs per sample. Median runtime was 3.963 h for BILBO and 3.180 h for nf-core/rnaseq, making BILBO 24.6% slower under this condition. Peak memory remained of a similar order (71.58 versus 66.77 GiB), whereas BILBO required substantially less temporary disk space (43.37 versus 135.45 GiB), corresponding to a 68.0% reduction. At 1 million read pairs, the observed times were 1.434 h for BILBO and 1.988 h for nf-core/rnaseq and peak disk increases were 44.05 and 64.60 GiB, respectively; however, the first nf-core run included initial workflow and plugin retrieval, so these values are reported as cold-start observations and are not used to claim a pure BILBO speed advantage. Technical repeatability at 5 million read pairs was high: the two BILBO count matrices were identical, and the two nf-core/rnaseq matrices had a median per-sample correlation of 0.9991.

Taken together, these results show that BILBO preserves the principal quantitative and biological conclusions obtained with a widely used reference workflow while offering a distinct computational profile. Its substantially lower temporary disk requirement represents a practical advantage for self-hosted installations and computational environments with limited storage capacity. The shorter runtime observed for nf-core/rnaseq defines a clear operational trade-off, positioning BILBO as a practical option when preserving storage capacity is more critical than minimizing wall-clock time.

### Functional annotation clustering framework enhances biological interpretation

**Fig. 3:**
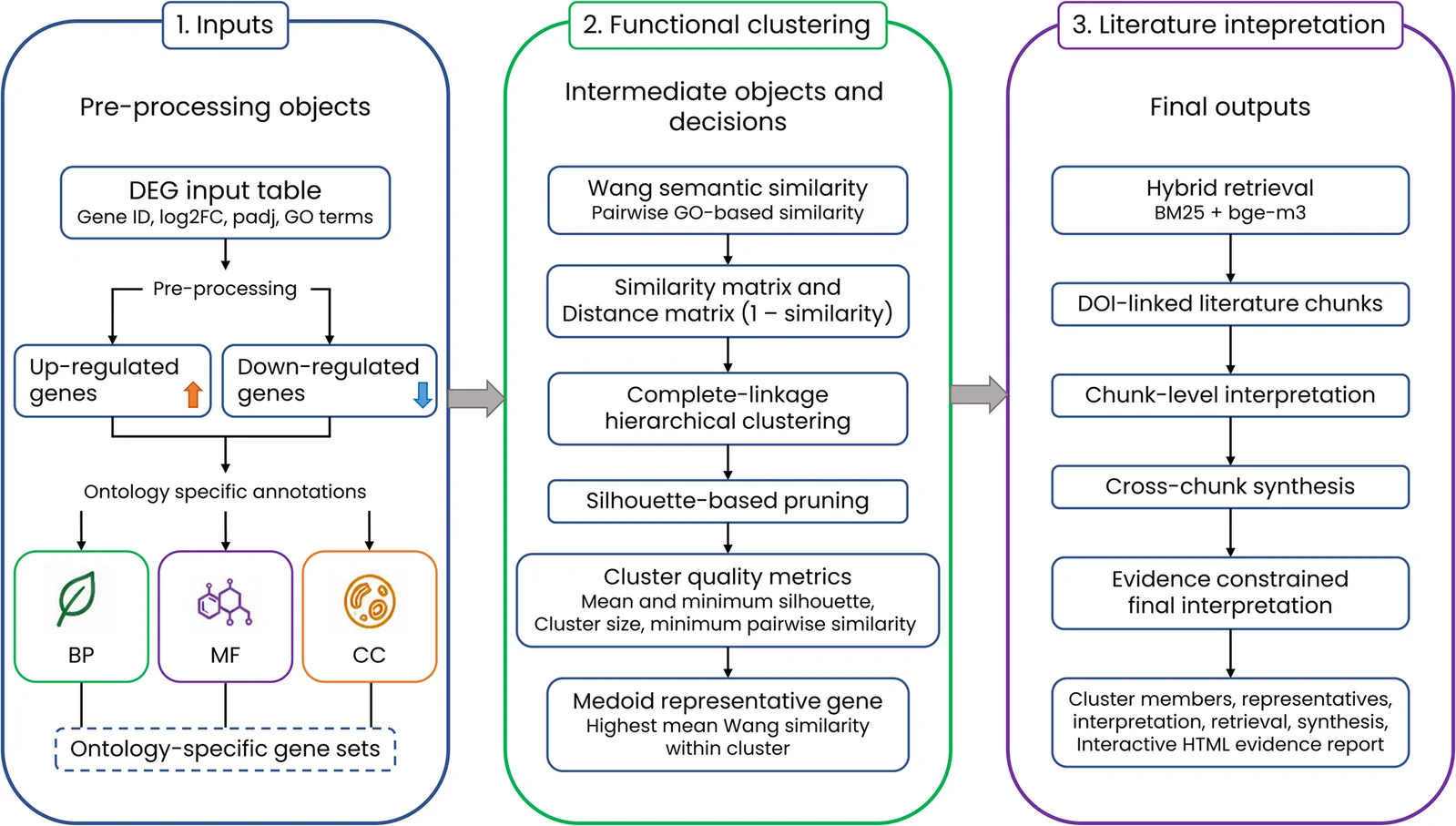
GO-based functional annotation clustering and RAG-assisted interpretation workflow. Differentially expressed genes are first separated according to regulatory direction and organized into ontology-specific annotation sets for biological process (BP), molecular function (MF), and cellular component (CC). Valid GO terms are used to compute Wang semantic similarity matrices, which are converted into distance matrices and submitted to complete-linkage hierarchical clustering. Cluster assignments are refined by silhouette-based pruning, and representative genes are selected as semantic medoids according to their centrality within each functional module. Representative genes are then submitted to the Retrieval-Augmented Generation (RAG) module, where hybrid retrieval combines BM25 and dense embeddings to identify DOI-linked literature chunks. Retrieved evidence is used for chunk-level interpretation, cross-chunk synthesis, and evidence-constrained final interpretation

The proposed functional annotation clustering strategy is designed to address a central limitation of conventional functional enrichment analyses: the fragmentation and redundancy of biological interpretation arising from large, unstructured lists of annotated genes. In BILBO, functional clustering operates as a post–differential expression analytical layer that organizes genes according to similarity in their functional annotation profiles, thereby enabling higher-level interpretation of transcriptomic results (Fig. 3).

BILBO defines gene similarity from the semantic structure of GO annotations. In this framework, cluster formation is based on how closely genes are related within the GO directed acyclic graph, rather than only on separation in a projected feature space. This connects each cluster directly to curated ontology relationships and preserves the distinction between biological process (BP), molecular function (MF), and cellular component (CC) annotations.

The clustering pipeline begins with rigorous preprocessing of functional descriptions derived primarily from GO annotations and complementary annotation sources generated during the RNA-Seq analysis. Before similarity calculation, GO identifiers are checked, and invalid or obsolete terms are removed from ontology-specific annotation sets. Up- and down-regulated genes are processed separately to avoid grouping genes with opposite expression patterns only because they share broad biological descriptors. BP, MF, and CC annotations are also clustered independently, so each ontology contributes a distinct functional view of the same DEG set. This ontology-specific organization keeps different types of functional evidence separated. BP clusters indicate shared participation in biological processes, MF clusters indicate related molecular activities, and CC clusters indicate similar cellular localization or compartment association. This allows users to inspect whether a functional signal is supported by process-level, activity-level, or localization-level evidence, while keeping the direction of regulation explicit.

Pairwise gene similarity is computed using the Wang semantic similarity measure over the GO directed acyclic graph. This method captures relationships between non-identical but related GO terms by considering their positions and semantic contributions within the ontology. Thus, genes annotated with related child or ancestor terms can still show high similarity, whereas genes sharing only broad or weakly informative annotations tend to be less strongly connected. The gene-level similarity matrix is converted into a distance matrix and used as input for agglomerative hierarchical clustering with complete linkage. Complete linkage was selected because it favors compact modules in which all members retain a minimum level of mutual similarity. This is useful for functional annotation clustering because it reduces the formation of large, weakly coherent groups caused by isolated similar pairs. As a result, retained clusters tend to represent consistent functional modules instead of loose collections of broadly related genes.

Cluster assignments are then refined through silhouette-based pruning. Genes with negative silhouette values are removed from their assigned clusters because their semantic distances indicate stronger agreement with another group. This step prevents weak assignments from inflating cluster size and helps preserve the functional consistency of the retained modules. Genes that cannot be assigned confidently remain in the output as unclustered, rather than being forced into a potentially misleading biological category. For each retained cluster, BILBO reports quality metrics that support direct inspection of the module before interpretation. These include individual silhouette values, mean silhouette, minimum silhouette, cluster size, minimum pairwise similarity, and a categorical cluster quality label. Mean silhouette summarizes cluster separation, minimum silhouette identifies the weakest retained member, and minimum pairwise similarity indicates the least similar gene pair within the module. Together, these metrics provide a transparent quality-control layer for the ontology-based clusters. Furthermore, a representative gene is selected for each functional module to provide a compact entry point for downstream interpretation. BILBO selects the semantic medoid of the cluster, defined as the real gene with the highest mean Wang similarity to the other members of the module. This strategy identifies the gene that is most central within the GO semantic structure of the cluster and is therefore suitable for literature retrieval and biological contextualization. When a representative gene is supported across more than one ontology, its relevance is reinforced by independent evidence from process, function, or localization annotations.

Both methods produced clusters in which MSigDB pathway sharing was substantially more frequent than in matched random groups. Among the 2,262 genes assigned by both methods, 453 were represented in KEGG_MEDICUS pathways. The mean proportion of gene pairs sharing at least one pathway was 69.8% for BILBO and 65.1% for the GOSemSim workflow, whereas the corresponding values in matched random groups were approximately 4%. The same pattern was observed among genes annotated with no more than 12 GO terms, for which the proportions were 62.0% and 57.6%, respectively. This indicates that the biological coherence recovered by BILBO was not dependent on the most extensively annotated genes.

The observed pathway-sharing proportion was 4.8% points higher for BILBO, with a 95% bootstrap margin of approximately ± 11.9% points. Thus, the two methods occupied the same overall performance range, with a higher observed value for BILBO. An additional over-representation analysis supported this interpretation. Hypergeometric tests were performed for each cluster, with Benjamini–Hochberg correction across the tested pathways. At least one MSigDB pathway was enriched at FDR < 0.05 in 17.6% of the BILBO clusters and 19.5% of the GOSemSim clusters. These comparable enrichment frequencies provide complementary evidence that both approaches recover biologically supported groups, while BILBO preserves a finer organization into compact functional clusters.

The sensitivity analysis showed that moderate changes to the GO similarity thresholds preserved the main cluster structure, with ARI values ranging from 0.87 to 0.96. Increasing the silhouette cutoff progressively removed uncertain assignments but did not reorganize the genes that remained clustered. Average linkage produced a more noticeable reorganization, whereas single linkage connected genes through long similarity chains and reduced the result to seven broad clusters. These results support the use of complete linkage and the silhouette filter to obtain compact functional groups.

By structuring genes into validated functional clusters, this approach transforms large, heterogeneous gene lists into compact functional units that are more amenable to biological interpretation. Rather than interpreting individual genes in isolation, researchers can reason at the level of functionally coherent groups, facilitating identification of dominant biological themes, representative genes, and pathways. This clustering strategy thus provides a principled and mathematically grounded mechanism for enhancing biological interpretation of RNA-Seq results, forming the conceptual foundation for downstream knowledge synthesis and automated interpretation within the BILBO platform.

### Automated biological interpretation through RAG

While the functional clustering module effectively organizes genes into biologically coherent groups based on GO semantic similarity, the subsequent step of biological interpretation remains a major bottleneck in high-throughput transcriptomics. Conventional LLMs, despite their fluency, are subject to factual hallucinations and “knowledge cutoffs,” rendering them unreliable for strict scientific deduction when used in isolation. The RAG implementation in BILBO addresses this by decoupling the model’s reasoning capabilities from its internal training data, dynamically connecting it to a curated corpus of scientific literature.

The interpretation workflow begins with a structured literature ingestion step. BILBO processes scientific articles by section and removes low-information fragments, including references, funding statements, methods-only passages, figure captions, tables, and table-like chunks, when they are unlikely to support functional interpretation. The remaining text is divided into sentence-aware chunks that preserve biological context across boundaries. Each chunk is stored with source metadata, including article title, section title, section index, chunk index, word count, and a stable chunk identifier, allowing each generated interpretation to be traced back to its supporting text. Retrieval is performed through a hybrid strategy that combines exact lexical evidence with semantic similarity. BM25 preserves sparse signals such as gene names, aliases, protein families, and ontology terms, which are essential when direct textual matches are available. Dense retrieval with BGE-M3 complements this search by recovering passages that may use different wording but still describe related biological processes, molecular functions, or phenotypic contexts. This is particularly useful in plant transcriptomics, where gene identifiers, aliases, protein family names, and functional descriptions often vary across species, databases, and annotation sources.

A critical distinction in our approach lies in the transition from GO-based functional clustering to literature-grounded interpretation. The sparse query is built from gene-centered metadata, including the primary gene name, GFF-derived name, UniProt gene names, parenthetical aliases, and compact symbol-like aliases. The dense query is built from the functional context associated with the representative gene, especially ontology-derived terms linked to its cluster. This separation prevents gene symbols from dominating semantic retrieval while preserving exact alias matching when direct evidence is available. Furthermore, the retrieval process is engineered to go beyond simple keyword matching through a semantic query expansion strategy. Dense and sparse results are fused using reciprocal rank fusion, and candidate chunks are reranked according to explicit biological evidence signals, including gene or alias matches, protein-family evidence, GO-term matches, and contextual biological terms. Source redundancy is controlled by limiting the number of chunks retrieved from the same article before diversifying across additional sources. This allows users to distinguish interpretations supported by several sources from interpretations based on repeated fragments from a single article.

The synthesis of this retrieved information is performed locally using an open LLM orchestrated through Ollama. At this stage, the model receives only the gene metadata, cluster-level functional context, and retrieved chunks. The system prompt imposes a closed-knowledge constraint, requiring biological claims to be derived from the provided fragments or explicitly marked as unsupported when the retrieved literature is insufficient. This converts the model into a constrained evidence synthesizer and reduces the risk of unsupported biological narratives.

Beyond generating descriptions, BILBO introduces a novel automated interpretation protocol that functions as an auditable review layer. The output is returned as a structured HTML, separating chunk-level observations, cross-chunk synthesis, and final interpretation. The chunk-level field describes what each retrieved passage supports, how it relates to the gene, the type of match involved, and any limitation of the evidence. The cross-chunk synthesis integrates only observations supported at the chunk level, while the final interpretation provides a concise biological statement with citation identifiers linked to the retrieved fragments. This structure allows users to inspect whether a conclusion is based on direct gene evidence, alias evidence, protein-family evidence, GO or process context, or weak background similarity. When the retrieved literature does not support a gene-specific conclusion, the model is instructed to state this limitation instead of extrapolating beyond the available evidence. Thus, the RAG module functions as an auditable, literature-grounded interpretation layer that supports hypothesis generation and expert evaluation while preserving transparency about the source and limitations of each biological claim.

Finally, the interpretation pipeline concludes with a targeted Representative Gene Analysis. Because representative genes are selected as semantic medoids of GO-based clusters, RAG queries are directed toward genes that occupy central positions within their functional modules. The model is prompted to describe the molecular or biological role of the representative gene, identify the type of retrieved evidence, and justify its use as a proxy for the cluster only when the literature fragments support this interpretation. In combination with functional annotation clustering, the RAG workflow provides a structured transition from differential expression results to biological interpretation. GO-based modules define coherent functional groups, semantic medoids provide meaningful retrieval targets, and evidence-constrained synthesis contextualizes these representatives using traceable literature fragments. This integration reduces the interpretive burden of large RNA-Seq outputs while keeping each generated interpretation linked to the evidence used to produce it.

Quantitative evaluation confirmed that the retrieval components contributed complementary strengths. Hybrid BM25 + BGE-M3 retrieval achieved the highest observed Precision@5 (0.862), compared with 0.815 for BM25 and 0.708 for BGE-M3, while its direct-evidence MRR was essentially identical to BM25 (0.511 and 0.510, respectively). BM25 produced the highest nDCG@10 (0.796) and pooled Recall@10 (0.488), compared with 0.785 and 0.469 for the hybrid method. Under the same protocol, BGE-M3 also exceeded PubMedBERT and BioBERT across all reported metrics; their Precision@5 values were 0.708, 0.369, and 0.262, respectively. Thus, hybrid fusion yielded the highest proportion of relevant passages among the first results presented to the interpretation workflow, while BM25 retained slightly greater ranking quality and pooled coverage.

Expert review was conducted for every claim retained in the final evaluation run. The workflow generated 11 claims for six of the 13 genes and withheld interpretation for seven genes when the available evidence was insufficient. Eight claims were fully supported, one was partially supported, two were unsupported, and none was contradicted by the cited literature. Strict claim accuracy was therefore 72.7%, increasing to 81.8% when partially supported claims were included, while citation correctness was 81.8%. In addition, 22 candidate claims were rejected by the deterministic evidence controls before presentation. Together, these findings show that the evidence controls favored supported, traceable statements and prevented unsupported specificity from being presented as biological interpretation.

### Overall discussion and biological relevance

The results presented in this study demonstrate that BILBO functions as an integrated platform capable of supporting complete RNA-Seq analyses while simultaneously addressing key challenges related to scalability, interpretability, and biological contextualization of transcriptomic data. By unifying data acquisition, processing, statistical analysis, and interpretation within a single self-hosted environment, the platform reduces fragmentation of analytical workflows and enables systematic exploration of RNA-Seq results across diverse experimental settings.

From a computational perspective, the modular architecture and asynchronous execution model allow complex RNA-Seq workflows to be executed in a controlled and traceable manner. The use of containerized execution environments ensures consistent behavior across analyses and facilitates deployment in heterogeneous computational infrastructures. These characteristics are particularly relevant for research groups that require reproducibility and data sovereignty without reliance on centralized web services or proprietary platforms. Also, the SEQC benchmark positions BILBO as a validated alternative to established RNA-seq workflows. Relative to nf-core/rnaseq, BILBO preserved the main quantitative and biological conclusions while requiring less temporary disk space, although with a longer warm-cache runtime. This trade-off may favor BILBO in self-hosted environments where storage capacity is a primary constraint.

The pathway analysis provides additional support for the biological coherence of the functional clustering strategy. Both BILBO and the GOSemSim workflow recovered substantially more MSigDB pathway sharing than expected in matched random groups, with a higher observed proportion for BILBO. This pattern was maintained among genes with fewer GO annotations, indicating that the result was not driven only by extensively characterized genes. Together with the sensitivity analysis, these findings support the use of complete linkage and silhouette filtering to generate compact functional groups while avoiding the chaining observed with single linkage.

Beyond technical integration, the biological relevance of BILBO emerges primarily from its ability to structure and contextualize differential expression results. Conventional RNA-Seq pipelines often culminate in extensive lists of differentially expressed genes and enriched functional terms, leaving the burden of interpretation to manual curation. The functional annotation clustering strategy introduced in this work transforms such outputs into compact, biologically coherent groups, enabling researchers to reason at the level of shared functional roles rather than isolated gene-level signals. This shift from gene-centric to function-centric interpretation facilitates the identification of dominant biological processes and reduces cognitive overload during exploratory analysis.

The combination of ontology-based semantic similarity, conservative hierarchical clustering, and evidence-constrained literature synthesis provides a principled balance between mathematical structure and biological interpretability. By using the GO directed acyclic graph as the basis for gene similarity, BILBO preserves the curated relationships among functional annotations while avoiding dependence on purely lexical or geometric proximity. Complete-linkage clustering and silhouette-based pruning favor compact and better-supported functional modules, whereas semantic medoid selection provides biologically meaningful representative genes for downstream interpretation.

The retrieval and expert-curation results further support the literature interpretation layer. Hybrid fusion produced the highest observed relevance among the first retrieved passages, which are those supplied to the synthesis model, while BM25 preserved complementary lexical coverage. At the interpretation stage, mandatory citations, explicit distinctions among gene-level, family-level, and general evidence, and deterministic rejection rules prevented 22 candidate claims from reaching the final output. The observed abstentions therefore reflect the intended behavior of the workflow when the available literature does not support a sufficiently cautious interpretation.

Collectively, these results indicate that BILBO not only automates RNA-Seq data processing but also enhances the interpretability of transcriptomic results through structured functional organization. The platform is therefore well suited for exploratory and hypothesis-generating studies, as well as for large-scale comparative analyses where consistent interpretation across multiple datasets is essential.

## Conclusions

In this work, we presented BILBO, a self-hosted platform designed to support large-scale RNA-Seq analyses through an integrated, reproducible, and interpretable computational framework. By consolidating data acquisition, preprocessing, statistical modeling, visualization, and interpretation within a single environment, BILBO addresses common limitations of fragmented RNA-Seq workflows and reduces the technical burden associated with managing complex bioinformatics pipelines.

A central contribution of this study is the introduction of a novel functional annotation clustering strategy that enhances biological interpretation of transcriptomic results. By combining rigorous GO-based semantic similarity, complete-linkage hierarchical clustering, silhouette-based pruning and semantic medoid selection, the proposed approach transforms extensive lists of differentially expressed genes into compact and biologically coherent functional groups. This structured representation enables higher-level reasoning about biological processes and facilitates exploratory analysis, while preserving a direct connection between each cluster and the underlying GO annotations. Coupled with literature-grounded RAG synthesis, this framework supports exploratory interpretation in a transparent and biologically constrained manner.

The integration of these methods within a modular, containerized architecture ensures that analyses remain reproducible, portable, and scalable across heterogeneous computational environments. Moreover, the platform’s emphasis on interactive visualization and structured output exploration allows users to transition seamlessly from quantitative results to biological insight, supporting both hypothesis generation and downstream experimental planning.

Overall, BILBO demonstrates that combining deterministic data structuring techniques with automated interpretation workflows can significantly improve the usability and interpretability of RNA-Seq analyses. The platform provides a practical contribution to computational life sciences by offering an open, extensible framework that supports end-to-end transcriptomic analysis while promoting biologically meaningful abstraction of complex genomic data.

## Supplementary Information

Below is the link to the electronic supplementary material.

Supplementary Material 1. Bioinformatics tools used in the BILBO RNA-seq pipeline, with their versions, corresponding pipeline stages, and fixed versus user-configurable parameters (DOCX 17.3 KB)

Supplementary Material 2. Controlled benchmarking of BILBO against nf-core/rnaseq using SEQC/MAQC-III. This document describes the benchmark design and execution environment and reports external TaqMan validation, expected-mixture reconstruction, cross-workflow count and differential-expression concordance, runtime, peak memory, peak temporary disk use, technical repeatability and study limitations at 1 and 5 million read pairs per sample (DOCX 24.5 KB)

Supplementary Material 3. List of scientific articles used to construct the plant RNA-seq literature corpus. Of the 1,671 studies identified in the PlantExp database, 1,612 were successfully retrieved and processed. The accompanying XLSX file contains two sheets: the first summarizes the plant species represented, including the number of studies, experiments, and total data volume (GB), and the second provides the complete list of DOIs for all included articles (XLSX 42.2 KB)

Supplementary Material 4. GO-based clustering and RAG summary tables. This XLSX file contains five sheets summarizing the functional clustering and RAG-assisted interpretation outputs: cluster members, cluster representatives, cluster-level interpretations, RAG retrieval evidence, and RAG synthesis. The file includes ontology- and direction-specific cluster assignments, representative genes selected for literature retrieval, summarized biological interpretations, retrieved DOI-linked evidence chunks, and expert review notes used to inspect the biological support of the RAG outputs (XLSX 139 KB)

Supplementary Material 5. Quantitative evaluation of the BILBO literature-retrieval and interpretation workflow. This document describes the evaluation design and reports Precision@5, nDCG@10, direct-evidence MRR, and pooled Recall@10 for BM25, BGE-M3, hybrid BM25+BGE-M3 retrieval, BioBERT, and PubMedBERT. It also reports expert assessment of the final atomic claims, citation correctness, answer coverage, abstention, and the number of candidate claims rejected before presentation.BGE-M3 (DOCX 32.7 KB)

Supplementary Material 6. Runtime benchmarking of BILBO-specific interpretation modules. This file summarizes execution times for the GO-based functional clustering and RAG-assisted interpretation modules across DEG datasets of different sizes. The benchmarking includes the clustering, representative-gene selection, retrieval, and LLM-based synthesis steps, providing practical information on the scalability of the BILBO-specific components introduced in this study (DOCX 16.2 KB)

Supplementary Material 7. Annotated datasheet. The data refers to the contrast between Leaf_Sun_C1 and Leaf_Shade_C1, obtained from pre-processed libraries available in SRA under BioProject ID PRJNA609253 (XLSX 140 KB)

## Data Availability

The data that supports the findings of this study are available from the corresponding author upon reasonable request.

## Code availability

Source code, a stepwise deployment guide, and supporting software documentation are available at https://github.com/Lucianov-TheFarmer/BILBO.
