# Supplementary material for: BILBO: a self-hosted platform for large-scale RNA-Seq analysis with a functional annotation clustering framework
Source: Funct Integr Genomics. 2026 Oct 29;26(1):306. doi: 10.1007/s10142-026-02091-9 (PMC13719831; doi:10.1007/s10142-026-02091-9)
Supplement: Supplementary file 1 [file 10142_2026_2091_MOESM1_ESM.docx]

**Supplementary Table S1.** Bioinformatics tools used in the BILBO RNA-seq pipeline, with their versions, corresponding pipeline stages, and fixed versus user-configurable parameters.

| **Tool** | **Version** | **Pipeline stage** | **Predefined pipeline parameters** | **User-configurable interface parameters** |
| --- | --- | --- | --- | --- |
| SRA Toolkit (fasterq-dump) | 3.1.1 | Sample download | fasterq-dump --progress --split-files --skip-technical -t <tmp_dir> -O <output_dir> <accession> | <accession>, <user_id> |
| FastQC | 0.12.1 | Pre-trimming QC | fastqc -o <output_dir> <input_file> | <input_file> = raw FASTQ file; <output_dir> = QC output folder |
| FastQC | 0.12.1 | Post-trimming QC | fastqc -o <output_dir> <input_file> | <input_file> = trimmed FASTQ file; <output_dir> = post-trim QC folder |
| Trimmomatic | 0.39 | Adapter trimming and read filtering | trimmomatic PE or SE; -threads <threads>; -phred33 or -phred64  Output file layout depends on paired-end or single-end mode | ILLUMINACLIP:<adapter_file>:<seed_mismatches>:<palindrome_clip_threshold>:<simple_clip_threshold>:<min_adapter_length>; SLIDINGWINDOW:<window_size>:<quality_threshold>; LEADING:<quality_threshold>; TRAILING:<quality_threshold>; MINLEN:<length>; optional CROP:<length>; HEADCROP:<length>; AVGQUAL:<quality_threshold>  Adapter selection comes from the UI, including custom adapter content when applicable |
| STAR | 2.7.11b | Genome indexing | STAR --runThreadN <threads> --runMode genomeGenerate --genomeDir <STAR_index> --genomeFastaFiles <genomic.fa> --sjdbOverhang <value>; --sjdbGTFfile <genomic.gtf> | <genome_dir>, <sjdb_overhang>, <threads> |
| STAR | 2.7.11b | Read alignment | STAR --runThreadN <threads> --genomeDir <STAR_index> --readFilesIn <read_1> <read_2> --outFileNamePrefix <prefix> --outSAMstrandField intronMotif --outSAMtype BAM Unsorted | <sample>, <genome_dir>, <threads>, --outFilterType=<value>, --outFilterMultimapNmax=<value>, --alignSJoverhangMin=<value>, --alignSJDBoverhangMin=<value>, --outFilterMismatchNmax=<value>, --outFilterMismatchNoverReadLmax=<value>, --alignIntronMin=<value>, --alignIntronMax=<value>, --alignMatesGapMax=<value> |
| Picard | 3.3.0 | Post-alignment cleanup | SortSam I=<input.bam> O=<sorted.bam> SORT_ORDER=queryname; AddOrReplaceReadGroups I=<input.bam> O=<rg.bam>; MarkDuplicates I=<input.bam> O=<dedup.bam> REMOVE_DUPLICATES=true M=<metrics.txt> | RGID=<sample_name> identifier for the read group; RGLB=lib1 library label; RGPL=ILLUMINA sequencing platform; RGPU=unit1 platform unit; RGSM=<sample_name> sample name.  In the current implementation, RGID and RGSM are derived from the sample basename, while RGLB, RGPL, and RGPU are fixed values |
| NCBI Datasets CLI | 18.0.2 | Genome search | datasets summary genome taxon <taxon> or datasets summary genome accession <accession> | <taxon> or <accession> search term |
| NCBI Datasets CLI + dataformat | 18.0.2 | Reference genome download | datasets download genome accession <accession> --filename <accession>.zip --include genome,gtf,gff3  Archive validation with unzip -tq; extracted files renamed to genomic.fa, genomic.gtf, genomic.gff3 | <accession> |
| htseq-count | 2.0.5 | Quantification | htseq-count -a 10 -t <feature_type> -i <id_attribute> -f bam --stranded=no <input.bam> <annotation_file> | <sample.bam>, <feature_type>, <id_attribute>, <selected_genome> |
| edgeR | 4.8.2 | Preprocessing and DEG | readTargets, readDGE, rowMeans >= 10 filtering, DGEList, calcNormFactors, estimateGLMCommonDisp, estimateGLMTrendedDisp, estimateGLMTagwiseDisp, glmFit, glmLRT, topTags  Export to DEG.xlsx | <preprocess_dir>, <deg_dir>, selected contrasts, selected genome accession |
