# Supplementary material for: BILBO: a self-hosted platform for large-scale RNA-Seq analysis with a functional annotation clustering framework
Source: Funct Integr Genomics. 2026 Oct 29;26(1):306. doi: 10.1007/s10142-026-02091-9 (PMC13719831; doi:10.1007/s10142-026-02091-9)
Supplement: Supplementary file 2 [file 10142_2026_2091_MOESM2_ESM.docx]

**Supplementary File S5. Controlled benchmarking of BILBO against nf-core/rnaseq using SEQC/MAQC-III**

**Benchmark scope and design**

BILBO was benchmarked against nf-core/rnaseq v3.26.0 as a mature, community-curated, reference-based RNA-seq workflow. The benchmark used 16 paired-end SEQC/MAQC-III libraries from GEO series GSE47774 (COH site): four replicates each of reference samples A (Universal Human Reference RNA) and B (Human Brain Reference RNA), and mixtures C (75% A + 25% B) and D (25% A + 75% B). Deterministic nested subsets of 1 and 5 million read pairs per sample were generated with a fixed seed. Acquisition and subsampling were shared and excluded from timed regions. Both workflows used the same Ensembl GRCh38 release 112 genome and annotation and were configured with the same ceilings of 128 logical CPUs and 128 GB RAM.

The comparison is intentionally end-to-end rather than a head-to-head test of identical algorithms. BILBO used FastQC, Trimmomatic, STAR, Picard and HTSeq-count; nf-core/rnaseq used the star_salmon route with Trim Galore and Salmon/tximport. Index generation, quality control, trimming, alignment, post-processing, quantification and report generation were included in wall time. Modules without a BILBO counterpart were disabled in nf-core/rnaseq. One technical run per workflow was performed at 1 million pairs per sample; two technical runs per workflow were performed at 5 million pairs, with workflow order reversed in the second repeat. Therefore, results are reported descriptively as observed values or median (range), without inferential testing across technical runs. The first nf-core run at 1 million pairs also recorded initial retrieval of the nf-core/rnaseq workflow source and the nf-schema plugin. Its runtime is therefore retained as a transparent cold-start observation, whereas comparative runtime interpretation is based primarily on the two warm-cache 5-million-pair runs.

**Supplementary Table S5.1.** Benchmark inputs, software, and execution environment.

| **Item** | **Specification** |
| --- | --- |
| Benchmark dataset | SEQC/MAQC-III; GSE47774; COH site; paired-end Illumina |
| Experimental structure | A, B, C and D; four replicates per material; 16 samples total |
| Mixture truth | C = 75% A + 25% B; D = 25% A + 75% B |
| Read depths | 1,000,000 and 5,000,000 paired fragments per sample; nested deterministic subsets |
| Reference | Homo sapiens GRCh38 primary assembly; Ensembl release 112 GTF |
| BILBO build | BILBO-rebuild corresponding to merged PR #6; expected commit 9c32396ca0a29173ccd7ae60b7b103637e5c156c |
| BILBO core | FastQC 0.12.1; Trimmomatic 0.39; STAR 2.7.11b; Picard 3.3.0; HTSeq 2.0.5; R 4.4.0 |
| Comparator | nf-core/rnaseq 3.26.0; star_salmon aligner route; Trim Galore; Docker profile |
| Compute limit | 128 logical CPUs and 128 GB RAM per workflow run |
| Hardware | 2 × AMD EPYC 7713; 256 logical CPUs available; 1.01 TB RAM; Linux x86_64 |
| Technical repeats | 1M: n = 1 per workflow; 5M: n = 2 per workflow with alternating order |
| External validation | TaqMan RT-PCR data from Bioconductor seqc 1.40.0; expected C/D mixture proportions |

**Computational performance**

**Supplementary Table S5.2.** End-to-end resource use. Values are observed values at 1M or median (range) at 5M; the nf-core 1M run was a cold start.

| **Depth** | **Workflow** | **n** | **Wall time (h)** | **Peak memory (GiB)** | **Peak disk increase (GiB)** |
| --- | --- | --- | --- | --- | --- |
| 1M | BILBO | 1 | 1.434 | 69.24 | 44.05 |
| 1M | nf-core/rnaseq | 1 | 1.988 | 67.11 | 64.60 |
| 5M | BILBO | 2 | 3.963 (3.958–3.968) | 71.58 (71.57–71.58) | 43.37 |
| 5M | nf-core/rnaseq | 2 | 3.180 (3.179–3.181) | 66.77 (66.50–67.04) | 135.45 (135.11–135.79) |

The observed 1M times were 1.434 h for BILBO and 1.988 h for nf-core/rnaseq; because the latter included initial workflow and plugin retrieval, this is a cold-start observation and is not used to claim a pure execution-speed advantage. In the replicated warm-cache 5M condition, BILBO required 24.6% more wall time. BILBO reduced observed peak disk increase by 31.8% at 1M and 68.0% at 5M; the replicated warm-cache 5M estimate is the primary disk comparison. Peak memory was similar in magnitude between workflows. CPU percentages are retained in the machine-readable archive but are not used as a primary endpoint because nf-core launches nested containers and aggregate Docker CPU percentages are not directly comparable with the single BILBO worker container.

**External biological validity**

**Supplementary Table S5.3.** External validity against TaqMan RT-PCR and known SEQC mixture proportions.

| **Depth** | **Metric** | **BILBO** | **nf-core/rnaseq** |
| --- | --- | --- | --- |
| 1M | TaqMan Spearman, A | 0.8147 | 0.8343 |
| 1M | TaqMan Spearman, B | 0.8076 | 0.8230 |
| 1M | TaqMan Spearman, C | 0.7205 | 0.7566 |
| 1M | TaqMan Spearman, D | 0.6944 | 0.7224 |
| 1M | TaqMan log2FC Spearman, A/B | 0.9617 | 0.9664 |
| 1M | TaqMan log2FC median absolute error, A/B | 0.3989 | 0.3962 |
| 1M | Mixture C Spearman | 0.9839 | 0.9832 |
| 1M | Mixture D Spearman | 0.9856 | 0.9847 |
| 1M | Mixture C log2 RMSE | 0.2420 | 0.2758 |
| 1M | Mixture D log2 RMSE | 0.2229 | 0.2560 |
| 5M | TaqMan Spearman, A | 0.8359 | 0.8712 (0.8712–0.8712) |
| 5M | TaqMan Spearman, B | 0.8270 | 0.8606 (0.8606–0.8606) |
| 5M | TaqMan Spearman, C | 0.7718 | 0.8217 (0.8217–0.8217) |
| 5M | TaqMan Spearman, D | 0.7523 | 0.8021 (0.8021–0.8022) |
| 5M | TaqMan log2FC Spearman, A/B | 0.9630 | 0.9670 (0.9669–0.9670) |
| 5M | TaqMan log2FC median absolute error, A/B | 0.3944 | 0.3882 (0.3851–0.3913) |
| 5M | Mixture C Spearman | 0.9951 | 0.9947 (0.9946–0.9948) |
| 5M | Mixture D Spearman | 0.9959 | 0.9954 (0.9953–0.9954) |
| 5M | Mixture C log2 RMSE | 0.1976 | 0.2093 (0.2072–0.2114) |
| 5M | Mixture D log2 RMSE | 0.1817 | 0.1998 (0.1982–0.2014) |

*Higher Spearman coefficients and lower MAE/RMSE values indicate stronger external agreement. The seqc TaqMan table contained 43 duplicate assay rows by gene symbol; quantitative values were collapsed by the median and detection calls by majority before comparison. This operation affected only the orthogonal validation layer and not RNA-seq counts or differential-expression results.*

**Cross-workflow agreement and technical repeatability**

**Supplementary Table S5.4.** Agreement between BILBO and nf-core/rnaseq differential-expression results.

| **Depth** | **Statistical method applied to both matrices** | **Technical runs** | **log2FC Spearman** | **DE-set Jaccard** | **Genes compared** |
| --- | --- | --- | --- | --- | --- |
| 1M | Common robust edgeR quasi-likelihood | 1 | 0.9956 | 0.8223 | 11,557 |
| 1M | BILBO PR #6 native edgeR likelihood-ratio test | 1 | 0.9961 | 0.8415 | 10,455 |
| 5M | Common robust edgeR quasi-likelihood | 2 | 0.9944 (0.9944–0.9944) | 0.8448 (0.8440–0.8457) | 15,646 |
| 5M | BILBO PR #6 native edgeR likelihood-ratio test | 2 | 0.9951 (0.9951–0.9951) | 0.8580 | 14,738 |

**Supplementary Table S5.5.** Gene-level count concordance before differential-expression modeling.

| **Depth** | **Technical run** | **Shared gene IDs** | **Median library-total ratio, BILBO/nf-core (range)** | **Median Pearson log2(CPM+1) (range across samples)** |
| --- | --- | --- | --- | --- |
| 1M | 1 | 63,140 | 0.7843 (0.7766–0.7910) | 0.9206 (0.9133–0.9294) |
| 5M | 1 | 63,140 | 0.7515 (0.7454–0.7554) | 0.9228 (0.9153–0.9326) |
| 5M | 2 | 63,140 | 0.7515 (0.7454–0.7554) | 0.9229 (0.9155–0.9327) |

All 63,140 gene identifiers were shared between standardized matrices. Lower BILBO library totals are consistent with a more conservative count-assignment/removal route than the Salmon/tximport route; therefore, absolute library totals should not be interpreted as an accuracy endpoint. At 5M, the two BILBO count matrices were byte-identical and had per-sample correlation r = 1.000. The two nf-core matrices were not byte-identical but had median per-sample correlation r = 0.9991 (range 0.9988–0.9994).
