# Supplementary material for: BILBO: a self-hosted platform for large-scale RNA-Seq analysis with a functional annotation clustering framework
Source: Funct Integr Genomics. 2026 Oct 29;26(1):306. doi: 10.1007/s10142-026-02091-9 (PMC13719831; doi:10.1007/s10142-026-02091-9)
Supplement: Supplementary file 3 [file 10142_2026_2091_MOESM5_ESM.docx]

**Supplementary File S3. Quantitative evaluation of the BILBO literature-retrieval and interpretation workflow**

**Benchmark scope and design**

The literature-retrieval component was evaluated using a frozen set of 13 gene queries and a corpus of 53,037 literature passages. Five retrieval strategies were compared under the same queries, corpus, retrieval depth, and post-processing procedure: BM25, BGE-M3, hybrid BM25+BGE-M3 retrieval, BioBERT, and PubMedBERT.

The ten highest-ranked passages from each method were pooled for each query, deduplicated, and presented without method or rank information for expert assessment. A domain expert evaluated 479 unique query-passage pairs using three relevance levels: direct evidence (grade 2), indirect or contextual evidence (grade 1), and irrelevant evidence (grade 0). The final pool comprised 37 direct, 187 indirect, and 255 irrelevant judgments.

Retrieval performance was summarized using Precision@5, graded normalized discounted cumulative gain at rank 10 (nDCG@10), reciprocal rank of the first direct-evidence passage (direct-evidence MRR), and pooled Recall@10. Precision and pooled recall considered direct and indirect evidence relevant, whereas direct-evidence MRR required grade 2. Recall is described as pooled because relevance was assessed within the combined outputs of the evaluated systems rather than exhaustively across the full corpus.

**Supplementary Table S3.1. Information-retrieval performance under the common evaluation protocol.**

| **Retrieval method** | **Precision@5** | **nDCG@10** | **Direct-evidence MRR** | **Pooled Recall@10** |
| --- | --- | --- | --- | --- |
| Hybrid BM25 + BGE-M3 | **0.862** | 0.785 | **0.511** | 0.469 |
| BM25 | 0.815 | **0.796** | 0.510 | **0.488** |
| BGE-M3 | 0.708 | 0.576 | 0.221 | 0.370 |
| PubMedBERT | 0.369 | 0.266 | 0.169 | 0.167 |
| BioBERT | 0.262 | 0.164 | 0.077 | 0.088 |

The hybrid strategy produced the highest observed Precision@5 and a direct-evidence MRR comparable to BM25. BM25 produced the highest nDCG@10 and pooled Recall@10. Thus, hybrid fusion yielded the highest proportion of relevant passages among the first retrieved results, whereas BM25 retained slightly higher overall ranking quality and pooled coverage. BGE-M3 outperformed the evaluated BioBERT and PubMedBERT representations under this protocol. BioBERT and PubMedBERT were evaluated using attention-mask mean pooling, L2 normalization, 512-token truncation, and cosine similarity, without retrieval-specific contrastive fine-tuning.

**Expert curation of generated interpretations**

The final interpretation workflow generated atomic claims with mandatory claim-specific citations. Deterministic controls verified citation identifiers, restricted unsupported direct-gene or orthology statements, distinguished evidence concerning the query gene from family-level or general evidence, checked species and experimental-condition attribution, and withheld interpretations when the retrieved literature was insufficient.

Every claim retained in the frozen evaluation run was examined against its cited evidence by a domain expert and classified as supported, partially supported, unsupported, or contradicted. Citation correctness was assessed separately. The workflow retained 11 claims for six of the 13 evaluated genes and abstained from interpretation for the remaining seven.

**Supplementary Table S3.2. Expert assessment of the claims retained by the evidence-controlled workflow.**

| **Expert assessment** | **Claims** | **Proportion** |
| --- | --- | --- |
| Supported | 8 | **72.7%** |
| Partially supported | 1 | 9.1% |
| Unsupported | 2 | 18.2% |
| Contradicted | 0 | **0.0%** |

**Supplementary Table S3.3. Claim support, citation correctness, answer coverage, and abstention.**

| **Evaluation measure** | **Result** |
| --- | --- |
| Fully supported claims | 8/11 (72.7%) |
| Supported or partially supported claims | 9/11 (81.8%) |
| Correct claim-citation associations | 9/11 (81.8%) |
| Genes receiving an interpretation | 6/13 (46.2%) |
| Genes for which the workflow abstained | 7/13 (53.8%) |
| Candidate claims rejected before presentation | 22 |

Most retained claims were fully or partially supported, no claim was contradicted by the cited literature, and more than four-fifths of the claim-citation associations were judged correct. Accuracy is presented together with answer coverage and abstention because the workflow intentionally withholds an interpretation when the available evidence does not support a sufficiently cautious statement. The rejection of 22 candidate claims before presentation further documents the contribution of the deterministic evidence controls.
