# Supplementary material for: BILBO: a self-hosted platform for large-scale RNA-Seq analysis with a functional annotation clustering framework
Source: Funct Integr Genomics. 2026 Oct 29;26(1):306. doi: 10.1007/s10142-026-02091-9 (PMC13719831; doi:10.1007/s10142-026-02091-9)
Supplement: Supplementary file 4 [file 10142_2026_2091_MOESM6_ESM.docx]

**Supplementary File S4.** Runtime benchmarking of BILBO-specific clustering and RAG modules.

| Dataset size | Number of DEGs | Number of valid GO-annotated genes | GO term validation / update time (s) | Wang similarity matrix time (s) | Hierarchical clustering time (s) | Silhouette pruning time (s) | Semantic medoid selection time (s) | Total clustering time (s) | Hybrid retrieval time (s) | Chunk reranking / filtering time (s) | LLM synthesis time (s) | Total RAG time (s) | Total BILBO-specific interpretation time (s) |
| --- | --- | --- | --- | --- | --- | --- | --- | --- | --- | --- | --- | --- | --- |
| Small | **168** | **94** | **0.016** | **8.432** | **0.009525376** | **0.002358414** | **0.048385025** | **8.508268815** | **9.276452775** | **0.04263414** | **231.1305415** | **241.1727945** | **241.1727945** |
| Medium | **252** | **130** | **0.257** | **18.476** | **0.012713215** | **0.004218585** | **0.054005916** | **18.80393772** | **8.494854987** | **0.016659141** | **98.30206515** | **107.374216** | **107.374216** |
| Large | **486** | **268** | **0.017** | **44.284** | **0.009911921** | **0.005237907** | **0.120542153** | **44.43669198** | **4.695914391** | **0.093785105** | **378.5985841** | **384.0647808** | **384.0647808** |
| Very large | **686** | **354** | **0.034** | **84.591** | **0.014374612** | **0.008537973** | **0.199581686** | **84.84749427** | **4.568927423** | **0.135180009** | **579.6887065** | **585.0740009** | **585.0740009** |

* Benchmarking was performed on a local workstation equipped with an AMD Ryzen 9 9700X processor, an NVIDIA GeForce RTX 5070 GPU, and 16 GB of system memory, running Ubuntu 26.04 LTS. CPU resources were primarily used for GO term validation, Wang semantic similarity matrix calculation, hierarchical clustering, silhouette pruning, and semantic medoid selection, whereas GPU acceleration was used when available for local LLM inference during RAG-assisted synthesis. Reported times correspond to wall-clock execution time for each module and include only the BILBO-specific functional clustering and RAG interpretation steps, not the upstream RNA-seq preprocessing, alignment, quantification, or differential expression analysis performed by established third-party tools.
